# A validated stability-indicating HPLC method for the simultaneous determination of pheniramine maleate and naphazoline hydrochloride in pharmaceutical formulations

**DOI:** 10.1186/1752-153X-8-7

**Published:** 2014-02-01

**Authors:** Taomin Huang, Nianzu Chen, Donglei Wang, Yonghua Lai, Zhijuan Cao

**Affiliations:** 1 Department of Pharmacy, Eye Ear Nose Throat Hospital of Fudan University, Fenyang Road, No. 83, Shanghai 200031, People’s Republic of China; 2 School of Pharmacy, Fudan University, Zhangheng Road, No. 826, Shanghai 201203, People’s Republic of China

**Keywords:** Liquid chromatography, Method validation, Pheniramine maleate, Naphazoline hydrochloride, Degradation products

## Abstract

**Background:**

A simple, rapid, and accurate stability-indicating reverse phase liquid chromatographic method was developed and validated for the simultaneous determination of pheniramine maleate and naphazoline hydrochloride in bulk drugs and pharmaceutical formulations.

**Results:**

Optimum chromatographic separations among pheniramine maleate, naphazoline hydrochloride and stress-induced degradation products have been achieved within 10 minutes by using an Agilent zorbax eclipse XDB C18 column (150 mm × 4.6 mm, 5 μm) as the stationary phase with a mobile phase consisted of 10 mM phosphate buffer pH 2.8 containing 0.5% triethlamine and methanol (68:32, v/v) at a flow rate of 1 mL min^-1^. Detection was performed at 280 nm using a diode array detector. Theoretical plates for pheniramine maleate and naphazoline hydrochloride were calculated to be 6762 and 6475, respectively. The method was validated in accordance with ICH guidelines with respect to linearity, accuracy, precision, robustness, specificity, limit of detection and quantitation. Regression analysis showed good correlations (*R*^2^ > 0.999) for pheniramine maleate in the concentration range of 150–1200 μg mL^-1^ and naphazoline hydrochloride in 12.5-100 μg mL^-1^. The method results in excellent separation of both the analytes and degradation products. The peak purity factor is ≥980 for both analytes after all types of stress, indicating complete separation of both analyte peaks from the stress induced degradation products.

**Conclusions:**

Overall, the proposed stability-indicating method was suitable for routine quality control and drug analysis of pheniramine maleate and naphazoline hydrochloride in pharmaceutical formulations.

## Introduction

**Figure 1:**
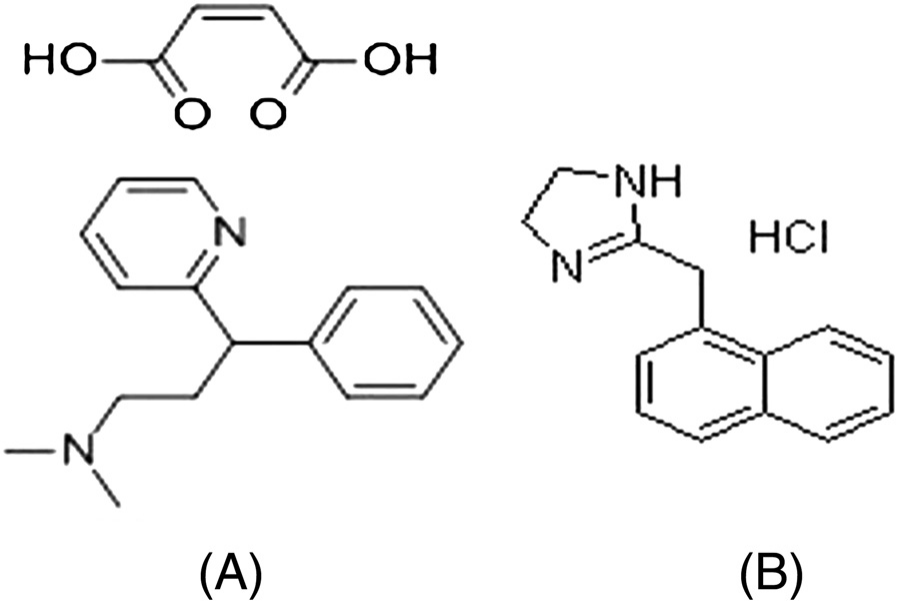
The structural formulae of (A) pheniramine maleate and (B) naphazoline hydrochloride.

Pheniramine maleate (*pKa* = 9.3), chemically known as N, N-Dimethyl-3-phenyl-3-(2-pyridyl) propylamine hydrogen maleate (C_16_H_20_N_2_⋅C_4_H_4_O_4_), is an antihistamine H_1_ receptor antagonist by inhibiting the effect of histamine on capillary permeability, gastric secretion, and contraction of bronchiolar and gastrointestinal smooth muscle [1,2]. Recently, Kerem Karaman et al. reported that pheniramine maleate could be used as an antihistaminic for the symptomatic relief of a hypersensitivity reaction, such as the acute ocular allergic reaction and short-duration, mild to moderate, intermittent ocular allergy [3]. Naphazoline hydrochloride (*pKa* = 10.8), chemically designated as 2-(1-naphthylmethyl)-2-imidazoline hydrochloride (C_14_H_14_N_2_⋅HCl), is a sympathomimetric agent with marked α-adrenergic activity and is a relatively long-lasting vasoconstrictor with a rapid action in reducing swelling when applied to the mucous membrane [4]. Figure 1 shows the structure formulae of pheniramine maleate and naphazoline hydrochloride. A novel fixed dose combination of pheniramine maleate and naphazoline hydrochloride is approved and available in the market, indicating that it can relieve redness, burning, irritation, and dryness of the eyes caused by wind, sun, and other minor irritants.

Various techniques have been reported for the determination of pheniramine maleate and naphazoline hydrochloride, respectively. Among them, the methods for the detection of pheniramine maleate included ultra-violet spectrophotometry [5], thin-layer chromatography-densitometry [6], and high performance liquid chromatography, etc. [7,8]. Naphazoline hydrochloride in pharmaceutical formulations or biological fluids either alone or in combination with other drugs was also detected by many methods, such as spectrophotometry [9,10], gas chromatography [11], high performance liquid chromatography [12-15], capillary electrophoresis [16-18], atomic emission and atomic absorption spectrometry [19], electrochemical method [20] and luminescence method [21,22]. However, in some extent, the above-described methods are limited in either low sensitivity or specificity. Furthermore, extensive survey revealed that no stability-indicating high performance liquid chromatography (HPLC) method has been reported including major pharmacopoeias such as USP, EP, JP and BP for the simultaneous determination of these two drugs in pharmaceutical formulation. Therefore, it is necessary to develop and validate a simple and accurate stability-indicating HPLC method for simultaneous determination of both drugs and their degradation products in pharmaceutical formulations.

## Results and discussion

### Optimization of the Chromatographic System

**Figure 2:**
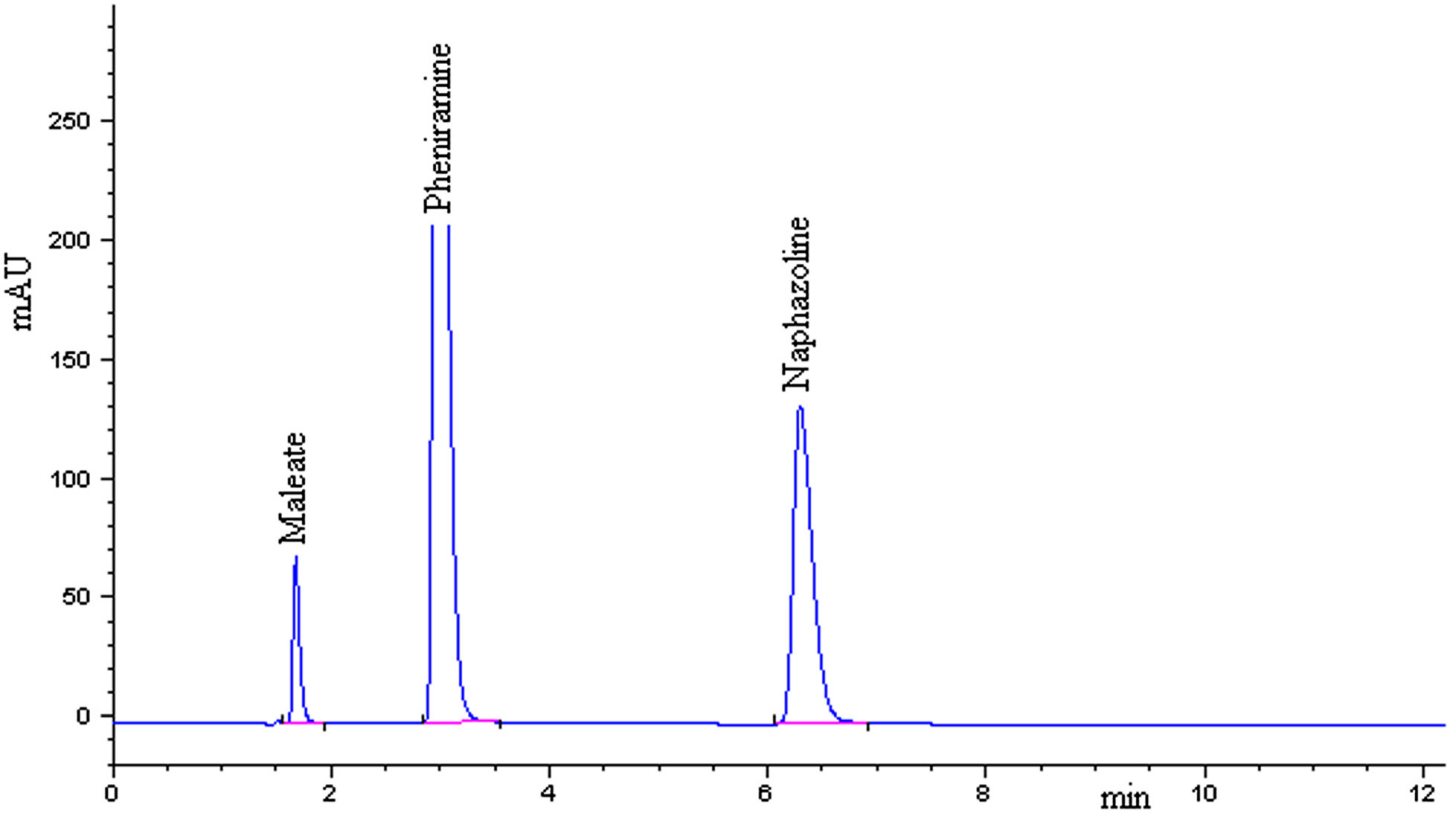
Chromatogram for separation of pheniramine maleate and naphazoline hydrochloride in pharmaceutical formulations.

There were two peaks of pheniramine maleate in the chromatography. Maleate is a dicarboxylic acid. Its ionization constant were *K*_1_ =1.0 × 10^-2^ and *K*_2_ = 5.5 × 10^-7^. Pheniramine maleate was ionized pheniramine positive ion and maleate negative ion in the mobile phase. The maleate negative ion was in the front (about 1.7 min) and pheniramine positive ion was behind the maleate peak (about 3.0 min) [Figure 2]. The content of pheniramine maleate was calculated according to the peak area of pheniramine (about 3.0 min) in this study.

The main objective of our work was to develop a stability-indicating HPLC method for determination of pheniramine maleate and naphazoline hydrochloride within a short run time between 3 to 10 min and symmetry between 0.80 and 1.20. The *pKa* of pheniramine maleate and naphazoline hydrochloride are 9.3 and 10.8 with a UV spectral maximum response at 262 nm and 280 nm, respectively. In the commercial eye drops in the market, the content of naphazoline hydrochloride (0.25 mg⋅mL^-1^) is far lower than that of pheniramine maleate (3 mg⋅mL^-1^). Therefore, the wavelength of 280 nm was used for LC detection.

Both pheniramine and naphazoline hydrochloride have high carbon to heteroatom ratio and have conjugated bond. Therefore, they can be separated through C18 stationary phase based mainly on their overall hydrophobicity. Pheniramine and naphazoline hydrochloride can also be separated using phenyl-Hexyl stationary phase considering their π electrons involving π-π interactions. Finally, both drugs also contain polar functional groups. So they may be separated using cyano stationary phase.

### Optimization of mobile phase, pH and stationary phase

The stationary and mobile phases play an important role on peak shape, symmetry, theoretical plates and resolution. To obtain symmetrical peaks with better resolution and no peak impurity, various chromatographic conditions was investigated and optimized for the determination of pheniramine maleate and naphazoline hydrochloride, such as mobile phase with different composition, pH and stationary phases with different packing material etc. That is, attempts were made by using three HPLC columns (Agilent zorbax eclipse XDB C18, Agilent Eclipse Plus Phenyl-Hexyl, and Dikma Platisil Cyano) with different mobile phase compositions and ratios. In all of the preceding columns, broad peaks were obtained for pheniramine and naphazoline hydrochloride by using different ratios (40:60, 50:50, 60:40, 70:30, 80:20) of methanol and water. No improvement of peak shape was obtained even when the temperature of column was enhanced to 40°C. The broad peak may be attributed to low polarity of the mobile phase. So different concentration (10 mM, 20 mM, 50 mM) of phosphate buffer was added to improve polarity of the mobile phase. The peak was narrowed but the peak symmetry was still not satisfactory. Then triethylamine (as silanol blocker) was added to the water phase, it was demonstrated that 10 mM phosphate buffer containing 0.5% v/v triethylamine was best for the improvement of peak shape. Moreover, buffer pH was found to be critical in the analytes separation and was extensively studied in method optimization. The effect of pH on retention was related with the ionization form of these solutes. In the attempt to investigate the effect of the mobile phase pH on the retention time and resolution of two substrates, the pH (2.8, 4.0, 5.0 and 6.0) was employed for this assay while keeping the other chromatographic parameters unchanged, i.e., Agilent zorbax eclipse XDB C18 column and the fixed mobile phase composition of 10 mM phosphate buffer containing 0.5% v/v triethlamine: methanol (68:32, *v/v*). As demonstrated in Table 1, a buffer pH of 2.8 was found to be optimal with narrow peak, resolution (R_s_ ≥ 3) and analysis time (t_R_ between 3 ~ 10 min), which was then selected for the following experiments.

**Table 1:** pH Optimization of phosphate buffer

**Mobile phase**	**Theoretical plates (N)**	**Symmetry**	**Resolution (R)**	**Peak shape**
**Methanol: Phosphate buffer, pH 2.8 (68:32)**				
Pheniramine	6762	0.91	7.75	+++
Naphazoline hydrochloride	6475	0.93		+++
**Methanol: Phosphate buffer, pH 4 (68:32)**				
Pheniramine	1319	0.26	4.13	___
Naphazoline hydrochloride	4737	0.43		+++
**Methanol: Phosphate buffer, pH 5 (68:32)**				
Pheniramine	6731	0.54	0.98	+++
Naphazoline hydrochloride	1325	0.24		___
**Methanol: Phosphate buffer, pH 6 (68:32)**				
Pheniramine	6499	0.54	2.75	+++
Naphazoline hydrochloride	1683	0.23		___

Following that, under the optimized mobile phase of methanol and 10 mM phosphate buffer with 0.5% *v/v* triethlamine (pH 2.8) (68:32, *v/v*), the experiments were performed on three different stationary phase. Highly symmetrical and sharp peaks of pheniramine and naphazoline hydrochloride were obtained on Agilent zorbax eclipse XDB C18 column (with better resolution, peak shapes, theoretical plates) as compared to other stationary phases (Agilent Eclipse Plus Phenyl-Hexyl, and Dikma Platisil Cyano), which was used in subsequent experiments.

### Method validation

The developed chromatographic method was validated using ICH guidelines [23,24]. Validation parameters included linearity, accuracy, precision, robustness, specificity, LOD and LOQ.

Linearity was verified by triplicate analysis of different concentrations. As a result, the linear regression equation was found to be *Y* = 5.0985*X* + 23.4917 (*R*^2^ = 0.9993, *n* = 6, 150–1200 μg mL^-1^) for pheniramine and *Y* = 28.3892*X* + 18.8795 (*R*^2^ = 0.9999, *n* = 6, 12.5-100 μg mL^-1^) for naphazoline hydrochloride, respectively. In which, Y was the dependent. variable, X was independent variable, 5.0985 and 28.3892 were slopes which showed change in dependent (Y) variable per unit change in independent (X) variable; 23.4917 and 18.8795 were the Y-intercept i.e., the value of Y variable when X = 0.

Based on a signal-to-noise ratio of 3:1, LOD was found to be 0.1 and 0.02 *μ*g mL^-1^ for pheniramine maleate and naphazoline hydrochloride, respectively. LOQ with a signal-to-noise of 10:1 was found to be 0.3 *μ*g mL^-1^ for pheniramine maleate and 0.07 *μ*g mL^-1^ for naphazoline hydrochloride, respectively.

Accuracy of the developed method was determined by analyzing samples before and after the addition of known amounts of pheniramine maleate and naphazoline hydrochloride. The acceptable recovery was set as between 97.0% and 103.0% [Table 2]. The developed analytical method had good accuracy with overall recovery rates in the range of 97.8%-102.1% for all compounds with RSDs below 1.3%, indicating that the proposed method was to be highly accurate and suitable for intended use.

**Table 2:** Accuracy of the proposed HPLC method

**Drugs**	**Spiked concentration (μg mL**^**-1**^**)**	**Measured concentration (μg mL**^**-1**^**) ± SD**	**Accuracy (%)**	**RSD (%)**
Pheniramine	150.0	151.2 ± 1.7	100.9	1.2
	600.0	612.4 ± 6.1	102.1	1.0
	1200.0	1187.6 ± 15.6	99.0	1.3
Naphazoline hydrochloride	12.5	12.6 ± 0.1	101.1	1.0
	50.0	49.8 ± 0.6	99.6	1.2
	100.0	97.8 ± 0.6	97.8	0.6

The precision was evaluated by analyzing the standard solutions of pheniramine maleate and naphazoline hydrochloride at three concentrations under the optimal conditions. It was considered at two levels: five times in one day for repeatability (intra-days) and on three consecutive days for intermediate precision (inter-days). The corresponding results were expressed as the relative standard deviation (RSD) and mean recovery of a series of measurements. The calculated RSD values of the intra- and inter-day assay were <1.0% and 1.2%, respectively [Table 3]. The results also demonstrated that pheniramine maleate and naphazoline hydrochloride were stable in solution.

**Table 3:** **Intra-day and Inter-day precision of the proposed HPLC method (*n*** **= 5)**

**Drugs**	**Actual concentration ( *μ *** **g mL**^**-1**^**)**	**Intra-day precision**	**Inter-day precision**
		**Measured concentration ± SD; RSD (%)**	**Measured concentration ± SD; RSD (%)**
Pheniramine	150.0	147.8 ± 1.1; 1.0	147.6 ± 1.3; 1.2
	600.0	608.5 ± 2.4; 0.4	607.0 ± 1.3; 1.0
	1200.0	1194.7 ± 2.7; 0.3	1197.6 ± 7.4; 0.6
Naphazoline hydrochloride	12.5	12.6 ± 0.05; 0.4	12.5 ± 0.1; 0.9
	50.0	50.3 ± 0.3; 0.5	50.4 ± 0.2; 0.4
	100.0	100.1 ± 0.4; 0.4	100.4 ± 0.5; 0.5

Robustness was validated by slightly varying the chromatographic conditions. In all of the deliberately varied chromatographic conditions (different flow rate, buffer composition and buffer pH), no obvious effect on the chromatographic parameters was observed [Tables 4 and 5].

**Table 4:** Robustness of pheniramine

**Chromatographic condition**	**Assay %**	**t**_**R **_**(min)**	**Theoretical plates**	**Symmetry**
Flow rate (0.9 mL min^-1^)	103.4	3.734	6623	0.89
Flow rate (1 mL min^-1^)	101.2	3.013	6762	0.90
Flow rate (1.1 mL min^-1^)	99.5	2..910	6745	0.91
Buffer: Methanol (70:30)	99.2	4.136	6612	0.87
Buffer: Methanol (68:32)	100.7	3.013	6523	0.88
Buffer: Methanol (65:35)	99.6	2.956	6524	0.86
Buffer (pH 2.6)	101.7	2.990	6456	0.92
Buffer (pH 2.8)	101.9	3.073	6346	0.85
Buffer (pH 3.0)	100.9	3.360	6678	0.86

**Table 5:** Robustness of naphazoline hydrochloride

**Chromatographic condition**	**Assay %**	**t**_**R**_** (min)**	**Theoretical plates**	**Symmetry**
Flow rate (0.9 mL min^-1^)	101.5	6.565	6534	0.91
Flow rate (1 mL min^-1^)	99.9	6.154	6745	0.93
Flow rate (1.1 mL min^-1^)	103.6	5.331	6521	0.88
Buffer: Methanol (70:30)	99.7	6.944	6346	0.90
Buffer: Methanol (68:32)	99.2	6.154	6351	0.89
Buffer: Methanol (65:35)	98.1	5.681	6566	0.86
Buffer (pH 2.6)	100.9	5.764	6812	0.85
Buffer (pH 2.8)	101.4	6.148	6745	0.89
Buffer (pH 3.0)	100.2	6.145	6625	0.87

**Figure 3:**
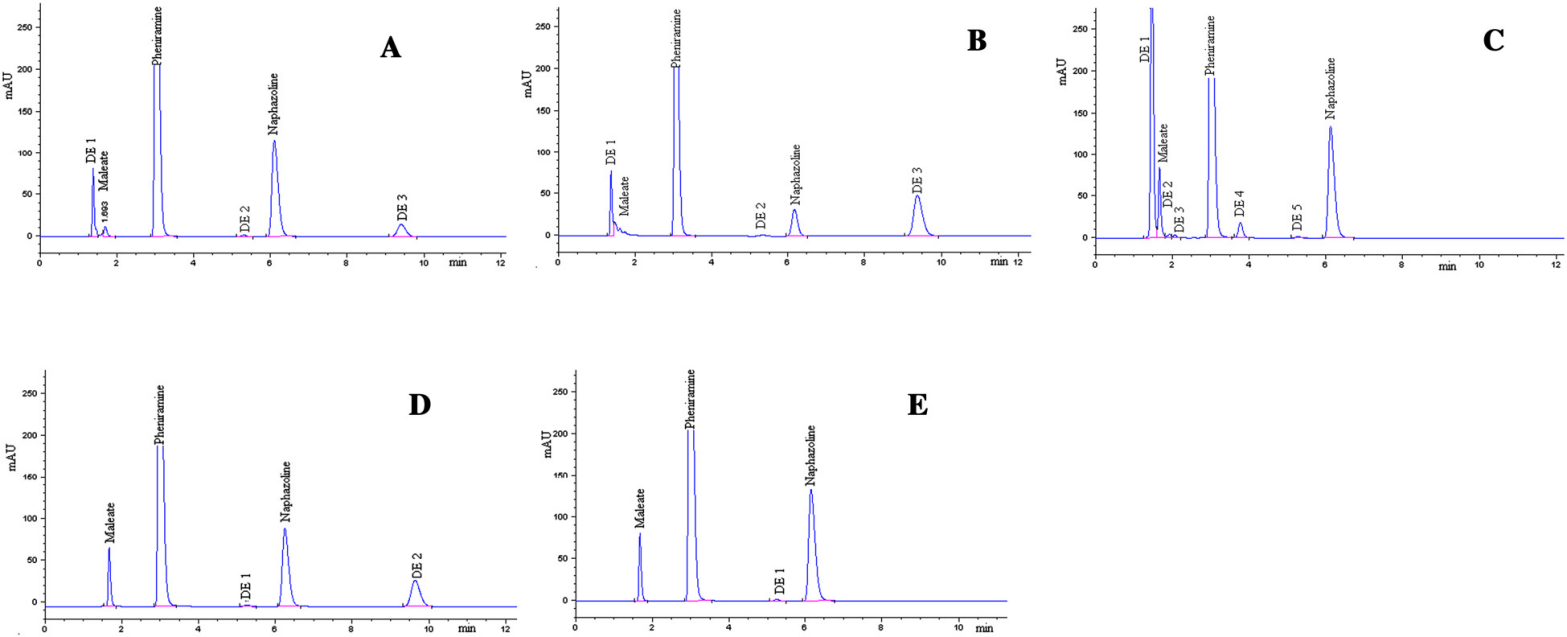
Chromatogram of pheniramine maleate and naphazoline hydrochloride under (A) acidic stress, (B) basic stress, (C) oxidative stress, (D) thermal stress and (E) photolytic stress.

Specificity was investigated by using photodiode array detection to ensure the homogeneity and evaluate purity of analyte peak. We found no interference of diluents and excipients firstly, and then the peak purity values were evaluated at different stress conditions (acid, base, oxidation, thermal and photolytic) for pheniramine maleate and naphazoline hydrochloride in formulation. As shown in Figure 3, several degradation products were detected, but had no influence on the main ingredients. The peak purity factor was more than 980 for drug product (Table 6), which further confirmed the specificity of this method.

**Table 6:** Stress testing results of pheniramine and naphazoline hydrochloride in stock solution

**Nature of stress**	**Storage conditions**	**Time (h)**	**Amount of pheniramine**	**Amount of naphazoline hydrochloride**	**Extent of decomposition**
			**Remaining ± SD (%)**	**Remaining ± SD (%)**	
5 M HCl	40°C	24	95.5 ± 0.9(PP = 999.213)	78.5 ± 3.8(PP = 999.412)	Substantial
5 M NaOH	40°C	2	65.3 ± 1.4(PP = 999.346)	44.9 ± 2.6(PP = 999.015)	Substantial
6% H_2_O_2_	40°C	24	84.9 ± 1.6(PP = 999.803)	76.0 ± 1.5(PP = 999.711)	Substantial
Thermal	40°C	120	98.2 ± 1.6(PP = 999.872)	102.5 ± 1.0(PP = 999.784)	None
	40°C	240	99.1 ± 1.3(PP = 999.423)	94.3 ± 1.2(PP = 999.651)	Substantial
Dry heat	105°C	7	99.2 ± 2.9(PP = 999.903)	68.4 ± 1.9(PP = 999.806)	Substantial
Photolytic	1.2 million lux hours and 200 W h/m^2^		98.2 ± 1.7(PP = 999.312)	98.5 ± 1.2(PP = 999.104)	None

*n* = Average of 3 determinations.

PP = peak purity factor, peak purity values in the range of 980 ~ 1000 indicate a homogeneous peak.

### Results of forced degradation study

All the stress conditions applied were enough to degrade two ingredients. Pheniramine was degraded up to 95.5% and naphazoline hydrochloride was degraded up to 78.5% when 5 M HCl was used. Pheniramine maleate was degraded up to 65.3% and naphazoline hydrochloride was degraded up to 44.9% when 5 M NaOH was used. Pheniramine maleate was degraded up to 84.9% and naphazoline hydrochloride was degraded up to 76.0% under oxidative stress. Pheniramine maleate was stable and naphazoline hydrochloride was degraded up to 94.3% for 240 h under thermal stress (40°C). Pheniramine maleate was stable and naphazoline hydrochloride was degraded up to 68.4% for 240 h under thermal stress (dry heat). Both pheniramine maleate and naphazoline hydrochloride were not degraded substantial under photolytic stress. From these stress studies it was thus concluded that pheniramine maleate and naphazoline hydrochloride were not stable in basic, acidic, oxidative and thermal conditions. The results of stress studies are shown in Table 6.

Aside from the percentage degradation of each ingredient, a number of degradation products (DE) were produced under acidic (3 degradation peaks with DE 3 as major degradation peak), basic (3 degradation peaks with DE 3 as major degradation peak), oxidative (5 degradation peaks with DE 1 as major degradation peak), thermal stress (dry heat) (2 degradation peaks with DE 2 as major degradation peak), photolytic stress (1 degradation peak). The developed method effectively separated the degradation products (3 degradation peaks under acidic stress, 3 impurity peaks under basic stress, 5 impurity peaks under oxidative stress, 2 impurity peaks under dry heat stress) from analyte peaks (Figure 3). Therefore, the developed method can be considered highly specific for intended use.

### Comparing the results with degradation of commercial eye drops sample solution

The degradation results of commercial eye drops sample solution were illustrated in Table 7. The stability results were in conformity with that in stock solution, which demonstrated that the developed method could be used to analyze pheniramine maleate and naphazoline hydrochloride in pharmaceutical formulation.

**Table 7:** Stress testing results of pheniramine and naphazoline hydrochloride in commercial eye drops sample solution

**Nature of stress**	**Storage conditions**	**Time (h)**	**Amount of pheniramine**	**Amount of naphazoline hydrochloride**	**Extent of decomposition**
			**Remaining ± SD (%)**	**Remaining ± SD (%)**	
5 M HCl	40°C	24	93.4 ± 0.7	80.2 ± 0.4	Substantial
5 M NaOH	40°C	2	61.3 ± 1.0	48.7 ± 1.6	Substantial
6% H_2_O_2_	40°C	24	87.8 ± 2.3	72.1 ± 1.8	Substantial
Thermal	40°C	120	99.2 ± 1.1	101.4 ± 1.4	None
	40°C	240	98.5 ± 1.4	93.3 ± 1.8	Substantial
Dry heat	105°C	7	98.6 ± 2.4	65.7 ± 2.5	Substantial
Photolytic	1.2 million lux hours and 200 W h/m^2^		97.6 ± 1.9	98.6 ± 2.3	None

*n* = Average of 3 determinations.

### Application of the developed method

Application of the developed method was checked by analyzing pheniramine maleate and naphazoline hydrochloride in commercially available pharmaceutical formulations. The results are provided in Table 8 which showed high percentage recoveries and low RSD (%) values for these two analytes.

**Table 8:** **Assay results of pheniramine maleate and naphazoline hydrochloride in commercial eye drops (*n*** **= 3)**

**Batch no.**	**Compounds**	**Labled**	**Found**	**RSD (%)**
10D09L	Pheniramine maleate	45 mg 15 mL^-1^	46.44 mg 5 mL^-1^	0.57
	Naphazoline hydrochloride	3.75 mg 15 mL^-1^	3.98 mg 15 mL^-1^	1.04
11F09H	Pheniramine maleate	45 mg 15 mL^-1^	46.05 mg 15 mL^-1^	0.20
	Naphazoline hydrochloride	3.75 mg 15 mL^-1^	3.90 mg 15 mL^-1^	1.09

## Conclusions

A rapid and efficient RP-HPLC method was developed for the estimation of pheniramine maleate and naphazoline hydrochloride in pharmaceutical formulation and their degradation products. The proposed method was demonstrated to be accurate, precise, specific, sensitive, linear and robust based on method validation. Satisfactory results were obtained in separating the peaks of active pharmaceutical ingredients from the degradation products produced by forced degradation. Furthermore, the new method are cost-effective without the requirement of ion pairing and other derivatization agents, which are tend to adsorb very strongly on the stationary phase, resulting in difficulty in recovering initial column properties. Overall, the method is stability-indicating and can be used for routine analysis in quality control and any kind of stability and validation studies.

## Methods

### Chemicals and reagents

All chemicals were analytical grade and used as received. All solutions were prepared in Milli-Q deionized water from a Millipore water purification system (Bedford, MA, USA). Pheniramine maleate was purchased from EDQM CS30026-F67081 Strasbourg, France. Naphazoline hydrochloride with stated purify of 99.2% (lot No.100111-201104) was purchased from the National Institute for the Control of Pharmaceuticals and Biological products, Beijing, China. Pheniramine maleate and naphazoline hydrochloride eye drops (claimed to contain 3 mg mL^-1^ pheniramine maleate and 0.25 mg mL^-1^ naphazoline hydrochloride) were obtained from S.A.Alcon-couvreur N.V., Rijksweg 14,2870, Puurs, Belgium (lot No. 10D09L,11F09H). The eye drops contains benzalkonium chloride as preservative in sterile aqueous base. Potassium dihydrogen phosphate (KH_2_PO_4_) was obtained from Sinopharm Chemical Reagent Co. Ltd. (Shanghai, China). Triethlamine (HPLC grade) was obtained from Fisher scientific (New Jersey, USA). Phosphoric acid was obtained from Lingfeng Chemical Reagent Co. Ltd. (Shanghai, China). HPLC- grade methanol was obtained from TEDIA (OH, USA). Mobile phase was filtered using 0.45 μm nylon filters from Millipore Co. (MA, USA).

### Equipment and chromatographic conditions

Samples were analyzed on an Agilent 1100 HPLC system (Agilent Technologies, Palo Alto, CA, USA), attached with a G1311A quaternary pump, a G1312A vacuum degasser, and a G1315B DAD detector. The detector wavelength was fixed at 280 nm and peak areas were integrated automatically using the Hewlett–Packard Chem Station software program. Other apparatus included an ultrasound generator and a SevenEasy pH meter (Mettler Toledo) that was equipped with a combined glass–calomel electrode. An electric-heated thermostatic water bath (DK-S28) and an oven (DGH-9203A) for thermal degradation were purchased from Shanghai Jing Hong Laboratory Instrument Co. Ltd (China). Photo stability studies were performed on a photo stability test chamber model Pharma 500-L (Weiss Technik UK Ltd., Germany). An Agilent zorbax eclipse C18 column (150 mm × 4.6 mm i.d., 5 *μ*m) was maintained at 30°C. The mobile phase was composed of a mixture 10 mM phosphate buffer (pH 2.8) containing 0.5% triethlamine and methanol in the ratio of (68:32, v/v). The flow rate of the mobile phase was set at 1 mL min^-1^. Measurements were made with 20 *μ*L of injection volume. For the analysis of forced degradation samples, the photodiode array detector was used in scan mode with a scan range of 200–400 nm. The peak homogeneity was expressed in terms of peak purity factor and was obtained directly from the spectral analysis report using the above-mentioned software.

### Preparation of standard solution

Standard stock solutions of pheniramine maleate (3 mg mL^-1^) and naphazoline hydrochloride (250 *μ*g mL^-1^) were prepared in water. Series working solutions were diluted to the desired concentration for accuracy, precision, linearity, solution stability and robustness etc.

### Preparation of sample solutions

Ophthalmic solution was transferred into a volumetric flask and diluted to requisite volume with mobile phase to obtain concentration equal to 600 *μ*g mL^-1^ of pheniramine maleate and 50 *μ*g mL^-1^ of naphazoline hydrochloride. The solution was filtered through 0.45 *μ*m nylon filter before analysis.

### Method validation

The proposed method was validated according to ICH guidelines [23] including specificity, accuracy, precision, LOD, LOQ, linearity, range and robustness.

Specificity was the ability of the method to measure the analyte from the excipients and potential impurities. The specificity of the developed method was investigated in the presence of degradation products.

The linearity test solutions were freshly prepared by diluting stock standard solutions with mobile phase. The linearity was tested at six levels ranging in 150–1200 *μ*g⋅mL^-1^ (150, 300, 450, 600, 900, 1200 *μ*g mL^-1^) for pheniramine maleate and 12.5-100 *μ*g⋅mL^-1^ (12.5, 25, 37.5, 50, 75, 100 *μ*g mL^-1^) for naphazoline hydrochloride. Each solution was prepared in triplicate. Calibration curves were plotted between the responses of peak versus analyte concentrations. The coefficient correlation, slope and intercept of calibration curve were calculated.

Accuracy of the developed method was determined by standard addition method. For this purpose, known quantities of pheniramine maleate (150, 600, 1200 *μ*g mL^-1^) and naphazoline hydrochloride (12.5, 50, 100 *μ*g mL^-1^) were supplemented to the sample solution previously analyzed. Then, the experimental and true values were compared [24].

The precision was tested by intra-day and inter-day precision at three level concentrations of standard mixture for pheniramine maleate (150, 600, 1200 *μ*g mL^-1^) and naphazoline hydrochloride (12.5, 50, 100 *μ*g mL^-1^). Intra-day precision was studied on the same day (*n* = 5). And inter-day precision was determined by performing the same procedures on three consecutive days. Percentage relative standard deviation (RSD%) for peak areas was then calculated to represent precision.

To determine the robustness of the developed method, the mobile phase composition, flow rate and pH of buffer solution were deliberately changed. The effect of these changes on chromatographic parameters such as retention time, symmetry and number of theoretical plates was then measured.

Limit of detection (LOD) and limit of quantitation (LOQ) values were determined at signal-to-noise (S/N) ratios of 3:1 and 10:1, respectively, by injecting a series of dilute solutions with known concentrations.

### Procedure for forced degradation study

Forced degradation was carried out using different ICH [25,26] prescribed stress conditions such thermolytic, photolytic, acid, base hydrolytic and oxidative stress conditions.

#### Acid degradation

For this purpose, 2 mL of the standard stock solution was transferred into a 10 mL volumetric flask. And then 2 mL 5 M HCl was added into the flask, which was kept at 40°C for 24 h in water bath [24]. After completion of the stress, the solution was cooled in room temperature and neutralized by using 5 M NaOH and the volume was completed up to the mark with mobile phase.

#### Alkali degradation

For this purpose, 2 mL of the standard stock solution was transferred into a 10 mL volumetric flask. Add 2 mL 5 M NaOH in the flask and keep at 40°C for 2 h in water bath. After completion of the stress, the solution was cooled in room temperature and neutralized by using 5 M HCl and diluted to the mark with mobile phase.

#### Oxidative degradation

For this purpose, 2 mL of the standard stock solution was transferred into 10 mL volumetric flask. Add 2 mL 6% H_2_O_2_ added in the flask and keep at 40°C for 24 h in water bath. After completion of the stress, the solution was cooled in room temperature and diluted to the mark with mobile phase.

#### Thermal degradation

Thermal degradation studies were performed at two different temperatures: 40°C in water bath and 105°C in oven (dry heat thermolysis). For thermal degradation at 40°C, 2 mL of the standard stock solution was transferred into 10 mL volumetric flask and kept at 40°C in water bath for 120 h and 240 h. After completion of the stress, the solution was cooled in room temperature and the volume was completed up to the mark with mobile phase. For dry heat thermolysis, 150 mg pheniramine maleate and 12.5 mg naphazoline hydrochloride were mixed in Petri dish at 105°C for 7 h. After completion of the stress, the powder mixture was dissolved and diluted to 50 mL with mobile phase. 2 mL of this solution was further diluted to 10 mL with mobile phase.

#### Photolytic degradation

Study was performed on dark control and photolytic exposed sample in a way to get the minimum exposure of 1.2 million lux hours for light and 200 W h/m^2^ for ultraviolet region.

### Degradation of commercial eye drops sample solution

The degradation of commercial eye drops sample solution was performed under the same above-mentioned stress conditions as the stock solution, including thermolytic, photolytic, acid, base hydrolytic and oxidative stress conditions.

## Abbreviations

HPLC: High performance liquid chromatography; DE: Degradation products.

## Competing interests

The authors declare that they have no competing interests.

## Authors’ contributions

TMH: Participate in method development and optimization, perform the experiments for forced degradation studies, collect experimental data and write the manuscript. ZJC: Propose and supervise the implementation of various experiments and write the manuscript. NZC, DLW and YHL: Participate in the experiments for method validation. All authors read and approved the final manuscript.
